# Supplementary material for: Structural basis of SNAPc-dependent snRNA transcription initiation by RNA polymerase II
Source: Nat Struct Mol Biol. 2022 Nov 24;29(12):1159–69. doi: 10.1038/s41594-022-00857-w (PMC9758055; doi:10.1038/s41594-022-00857-w)
Supplement: Source Data Fig. 1 — Unedited raw images of all the gels used in Fig. 1. All three replicates of gels for Fig. 1d are also furnished as used for quantification and statistical analysis in Fig. 1e [file 41594_2022_857_MOESM4_ESM.pdf]

Figure 1-a

M SNAPc-FL

M SNAPc-core

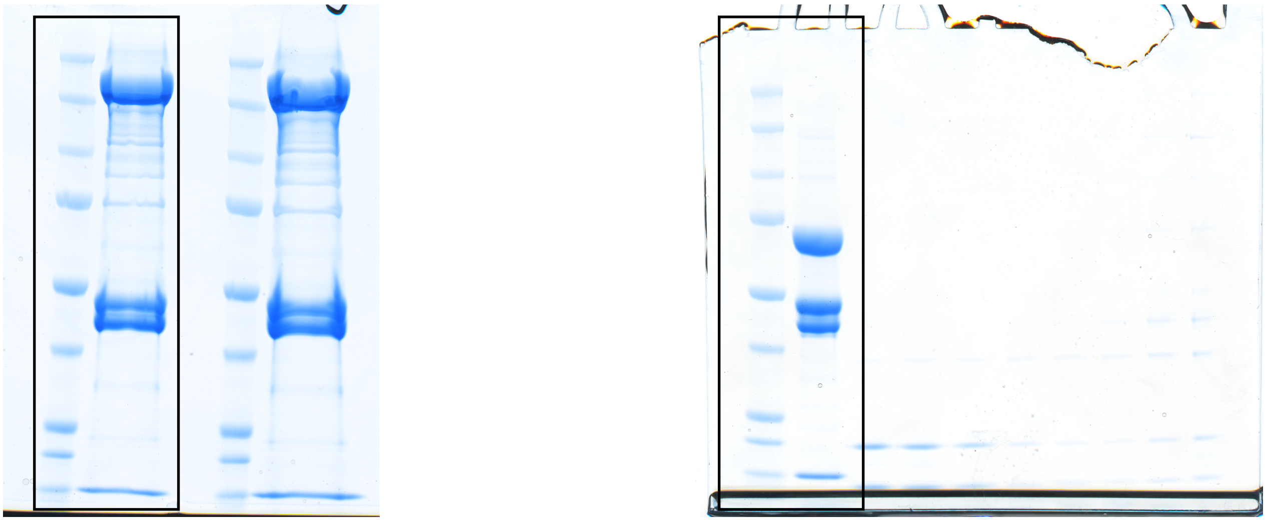

Figure 1-c

SNAPc-FL

SNAPc-core

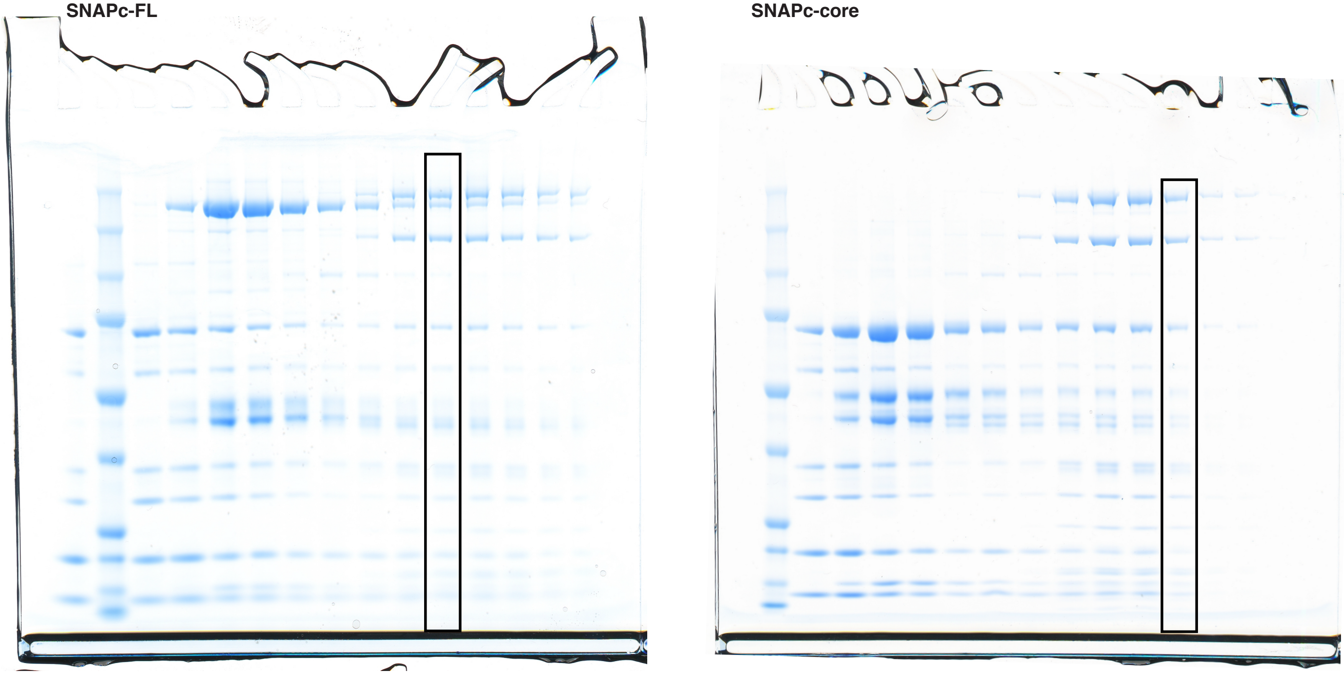

Figures: Source data of SDS_PAGE gels presented in Figures 1a, 1c. Black boxes represent the lane used in the in the respective figure.

Figure 1-b

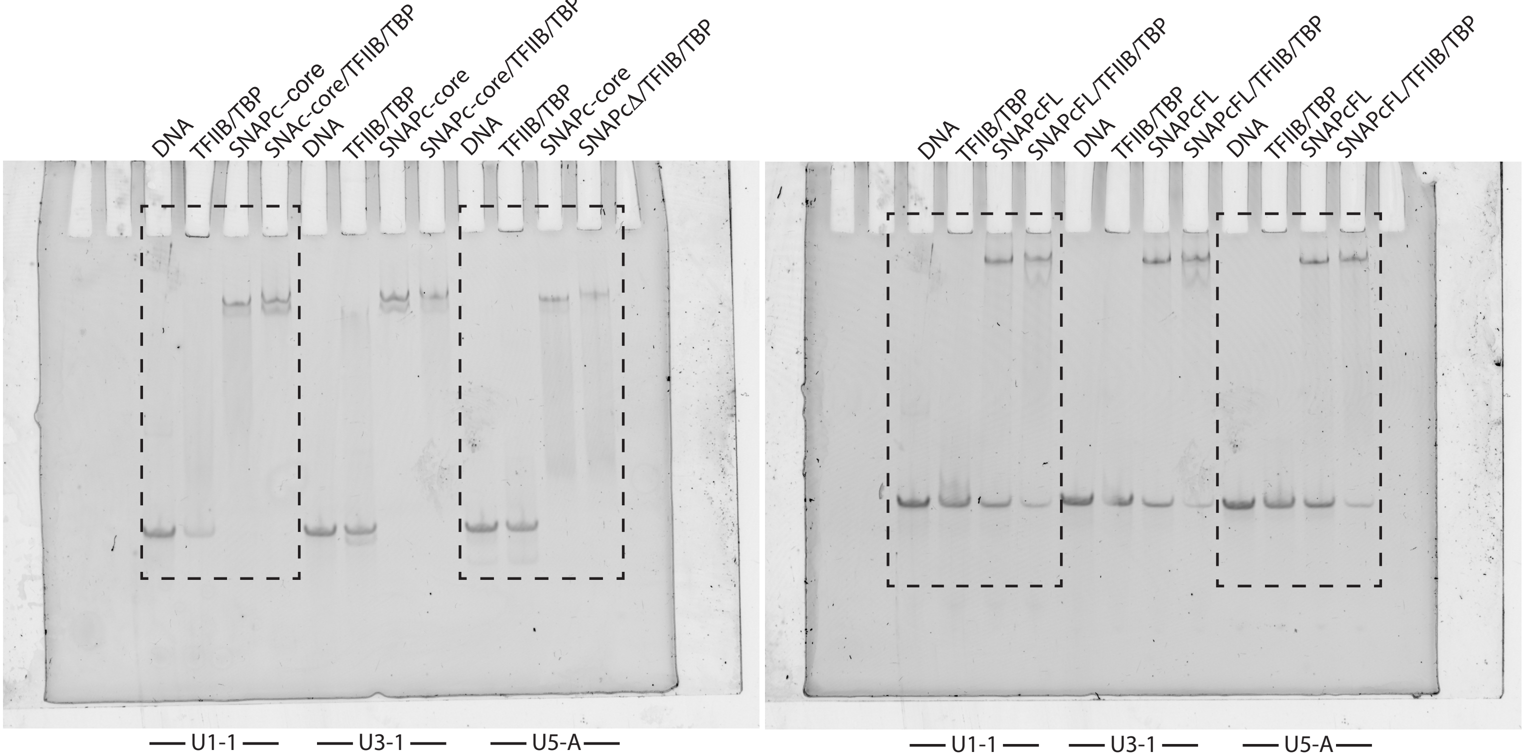

Figures: Source data of SDS_PAGE gels presented in Figures 1b. Dashed boxes represent the lanes used in the in the figure.

Replicate 1:

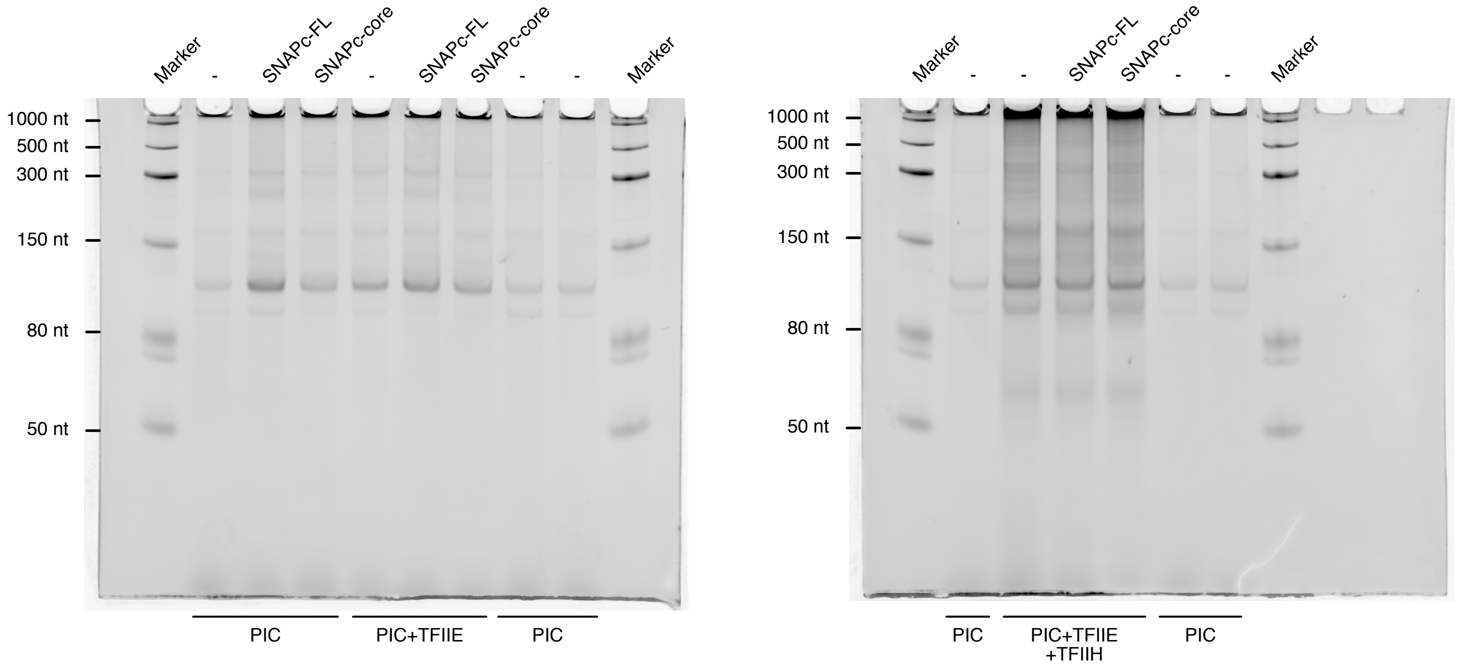

Replicate 2:

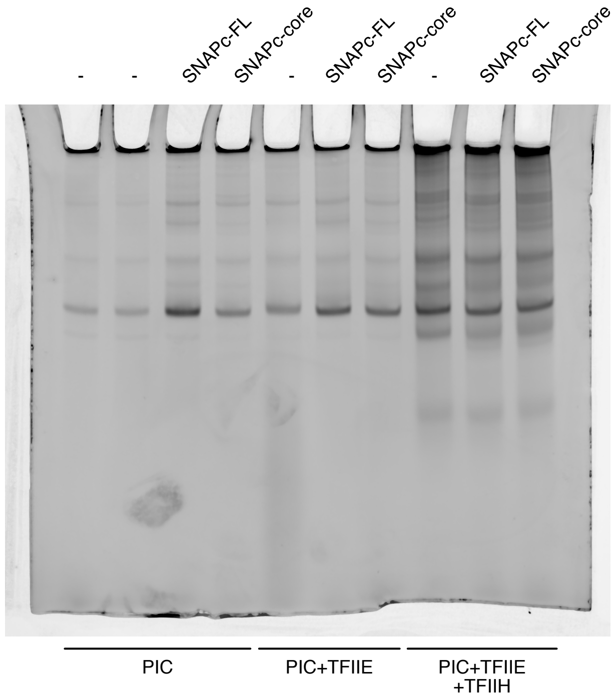

Replicate 3:

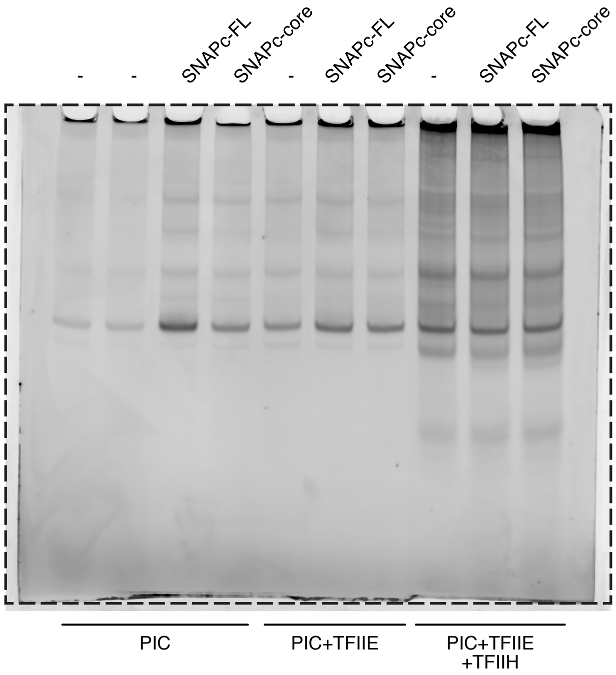

Figures: Source data of Urea-PAGE gels presented in Figures 1d, and the three replicates used for quantification in Figure 1e. Replicate 3 used in Figure 1d is highlighted in a dashed black box.
